# Heterotaxy polysplenia syndrome with cholangiopancreatic cancer: a case report and literature review

**DOI:** 10.3389/fmed.2025.1537227

**Published:** 2025-06-09

**Authors:** Ziye Chen, Yu-Ann Chen, Xuedong Wang, Wenyan Song, Yadi Geng, Pengfei Wang, Siming Kong, Jiahong Dong, Hui Bai, Yunfang Wang

**Affiliations:** ^1^School of Clinical Medicine, Beijing Tsinghua Changgung Hospital, Hepato-Pancreato-Biliary Center, Tsinghua University, Beijing, China; ^2^Center for Clinical and Translational Science, Beijing Tsinghua Changgung Hospital, Tsinghua University, Beijing, China; ^3^Precision Hepatobiliary Center, Beijing Tsinghua Changgung Hospital, Tsinghua University, Beijing, China; ^4^Hepatopancreatobiliary Center, School of Clinical Medicine, Beijing Tsinghua Changgung Hospital, Tsinghua University, Beijing, China; ^5^Faculty of Environment and Life, Beijing University of Technology, Beijing, China; ^6^Institute for Organ Transplant and Bionic Medicine, Tsinghua University, Beijing, China

**Keywords:** heterotaxy polysplenia syndrome, congenital pancreatic insufficiency, COVID-19, duodenal atresia, cholangiopancreatic cancer

## Abstract

**Background:**

Heterotaxy polysplenia syndrome is a rare condition characterized by multiple abnormal spleens and irregular placement of various organs. Some patients have been documented as developing various types of cancers, although the association with heterotaxy polysplenia syndrome remains uncertain. Most cases of heterotaxy polysplenia syndrome are isolated anomalies, but there are rare instances where it appears in closely related individuals, suggesting possible genetic inheritance.

**Case presentation:**

We report the case of a 36-year-old woman with heterotaxy polysplenia syndrome, who presented with choledocholithiasis, thickening and stenosis of the hepatic portal bile duct wall, duodenal atresia, and congenital pancreatic insufficiency. Following recovery from a COVID-19 infection, the patient incidentally identified a mass in the upper abdomen. Pathological examination revealed the mass to be a poorly differentiated adenocarcinoma of pancreaticobiliary origin. Additionally, a familial pattern of situs inversus and breast cancer was observed. In this report, we also provide a comprehensive literature review to enhance the understanding of heterotaxy polysplenia syndrome and to explore the potential association between this condition and tumor development.

**Conclusion:**

Our report contributes to the current knowledge about heterotaxy polysplenia syndrome and its potential association with tumors. Effective integration of clinical diagnostic information, exploration of precise means of diagnosing and treating rare diseases, and incorporation of the patient's familial background are promising for future research and clinical interventions.

## 1 Introduction

Polysplenia syndrome, a rare heterotaxy variant with an incidence of 1:250,000 live births, is marked by multiple splenules and cardiac and abdominal organ malformations. It presents as situs inversus (SI) or situs ambiguus (SA), reflecting organ inversion or misplacement, respectively (1–3). Although mostly sporadic, cases within related individuals suggest a genetic component, particularly when associated with conditions like primary ciliary dyskinesia or chromosomal disorders (4). Several genes, including ZIC3, NODAL, LEFTY2, and CFC1, have been implicated in the establishment of left-right asymmetry during organogenesis. Mutations in these genes can lead to abnormalities in the formation of the embryonic axis, which in turn affects the development of multiple organ systems (5). Patients exhibit a broad symptom spectrum with severe cases potentially leading to childhood mortality. The syndrome's phenotypic variability and potential severity highlight the complexity of its genetic basis. The prior reports have documented 14 cases of malignant tumors among patients with the syndrome from 1984 to 2014 (6), which underscores the necessity for research into the cancer risk associated with the syndrome, particularly in the context of genetic inheritance.

We here present a 36-year-old woman with adult heterotaxy polysplenia syndrome, characterized by an interrupted inferior vena cava (IVC), absence of the inferior retrohepatic vein, duodenal obstruction, and congenital pancreatic insufficiency. After recovering from a COVID-19 infection, the patient incidentally discovered an abdominal mass, which was pathologically confirmed as a poorly differentiated pancreaticobiliary adenocarcinoma. The family history indicates that the patient's mother has a history of breast cancer, and her sister is affected by situs inversus. Our review of the literature on polysplenia syndrome and its association with oncogenesis has deepened the understanding of this condition and its link to the risk of cancer genesis. It is expected to provide valuable insights for the clinical diagnosis and therapeutic intervention of such patients.

## 2 Case presentation

A 36-year-old female patient was admitted to our hospital with a clinical diagnosis of portal vein thrombosis, intrahepatic bile duct dilatation, and pancreatic space-occupying lesion. She reported a 2-month history of diminished appetite, postprandial abdominal distension, and generalized fatigue. A liver computed tomography angiography (CTA) and magnetic resonance cholangiopancreatography (MRCP) were performed at a local hospital. The computed tomography (CT) scan indicated the absence of a retrohepatic inferior vena cava, thrombosis of the left branch of the portal vein, intrahepatic bile duct dilatation, duodenum compression by an enlarged pancreatic head, and a diagnosis of polysplenia syndrome. A subsequent gastroscopy confirmed narrowing of the duodenal bulb. Following recovery from a COVID-19 infection, the patient incidentally detected a mass in the upper abdomen. She presented to our emergency department due to an inability to ingest food or water, resulting in significant abdominal distention, as well as symptoms of nausea and vomiting, seeking further diagnostic evaluation and therapeutic management.

**Figure 1:**
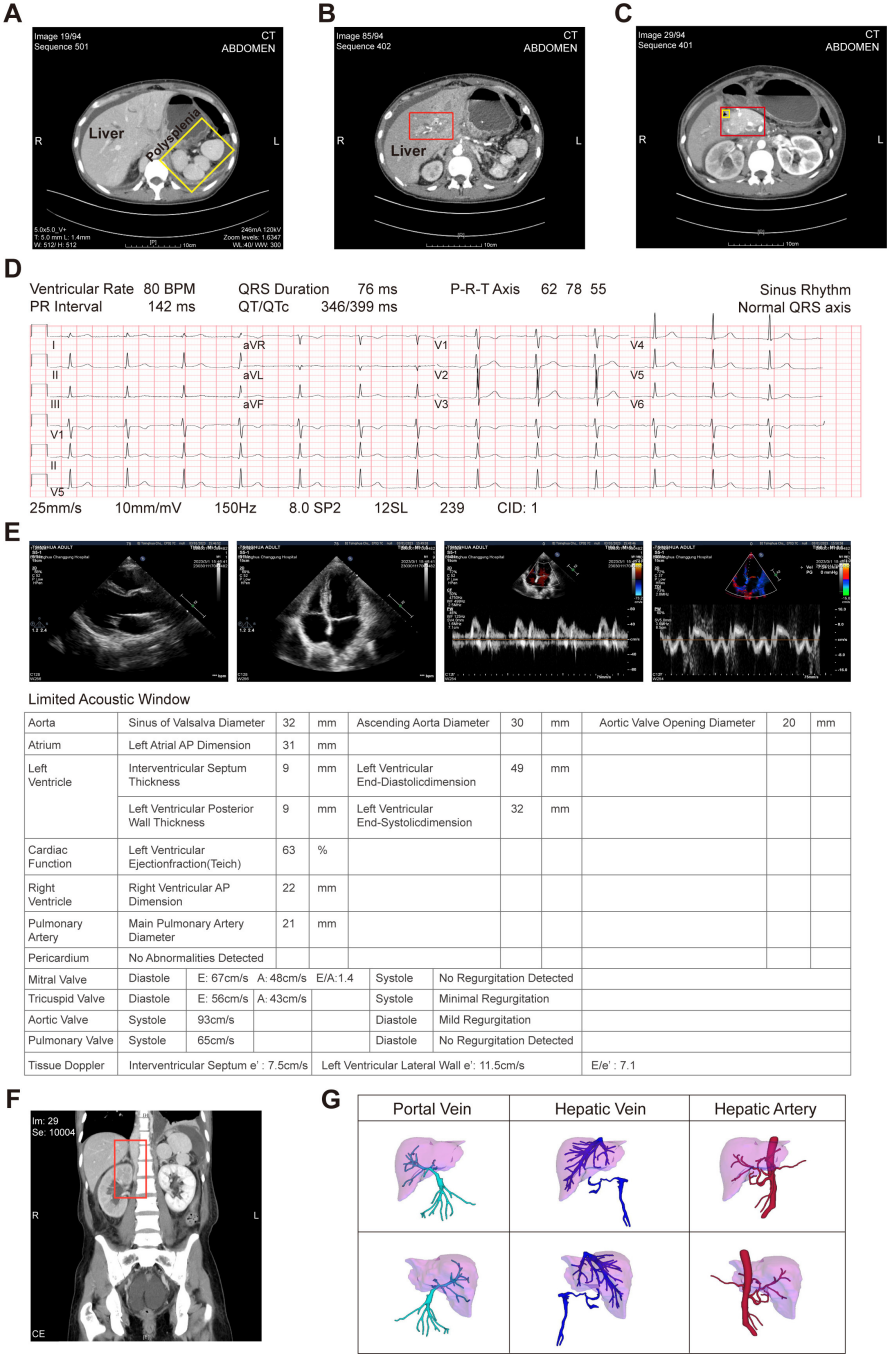
The enhanced computed tomography (CT) imaging of the patient's organ abnormalities and a comprehensive evaluation of cardiac and major vessel anatomy. **(A)** Multiple accessory spleens are indicated by the yellow box; **(B)** hepatopancreatic duct wall thickening and stenosis is denoted by the red box; **(C)** the enlarged pancreatic head (red box) is associated with duodenal atresia and obstruction, as highlighted within the yellow box; **(D)** the multilead electrocardiogram (ECG) revealed a normal sinus rhythm without any electrical axis deviation; **(E)** the echocardiographic (ECHO) revealed that all chamber dimensions are within normal limits. The ventricular wall thicknesses are normal across all segments, exhibiting coordinated movement and exhibiting normal systolic excursion. Resting wall motion was without evident segmental abnormalities. The left ventricular ejection fraction is normal. Valvular morphology and motion are unremarkable without significant dysfunction. Aortic and pulmonary arterial diameters are within the normal range. No obvious liquid dark area was detected in the pericardial cavity; **(F)** The enhanced CT scans depicted an occlusion of the inferior vena cava, denoted by the red box; **(G)** Three-dimensional hepatic imaging reveals the following anatomical variations: the portal vein bifurcates into left and right branches, with the left branch appearing attenuated and indistinct; The common hepatic artery exhibits anatomic variation, originating from the superior mesenteric artery, as does the splenic artery; The proper hepatic artery divides into left and right branches; Additionally, there is an absence of the right posterior inferior vena cava.

Upon admission, the initial physical examination of the patient revealed mild jaundice affecting the skin and mucous membranes. A mass approximately 4 by 5 cm was palpable in the upper abdomen, located just below the right costal margin. The examination did not detect a palpable spleen and there was no tenderness elicited over the hepatic or splenic regions. These physical findings, in conjunction with the CT imaging results, indicate the absence of splenic enlargement or other splenic structural abnormalities. Subsequent diagnostic evaluations confirmed the patient's diagnosis of polysplenia syndrome, congenital pancreatic insufficiency, and duodenal compression with associated stenosis and obstruction (Figures 1A–C).

Further evaluation of the patient's cardiac and major vascular anatomical structures and functional status was performed. The multilead electrocardiogram (ECG) revealed a normal sinus rhythm without any electrical axis deviation (Figure 1D). The echocardiographic (ECHO) assessment demonstrated unremarkable intracardiac structures and blood flow dynamics. The left ventricle exhibited preserved global systolic function, with no apparent diastolic dysfunction (Figure 1E). The patient did not undergo further CT pulmonary angiography or cardiac MRI, precluding direct visualization to determine the presence of pulmonary artery stenosis, atresia, or abnormal pulmonary venous drainage. However, the ECHO results suggested that the patient's aortic and pulmonary artery internal diameters were normal (21 mm), with no abnormally elevated systolic pulmonary valve flow velocity (65 cm/s). Additionally, the tricuspid regurgitant flow velocity indicated no abnormal right ventricular pressure (peak *E* wave 56 cm/s, peak *A* wave 43 cm/s), suggesting the absence of pulmonary artery stenosis or atresia. The anteroposterior diameter of the left atrium indicated a normal left atrial volume status (31 mm), partially suggesting no abnormality in pulmonary venous drainage (Figure 1E). The abdominal region imaging results indicated severe anomalies in the patient's hepatic vascular anatomy. The enhanced CT scans depicted an occlusion of the inferior vena cava (Figure 1F). Additionally, the three-dimensional imaging analysis indicated a constricted and indistinct left branch of the portal vein, an anomalous hepatic arterial configuration, and the absence of the right posterior inferior vena cava (Figure 1G).

**Figure 2:**
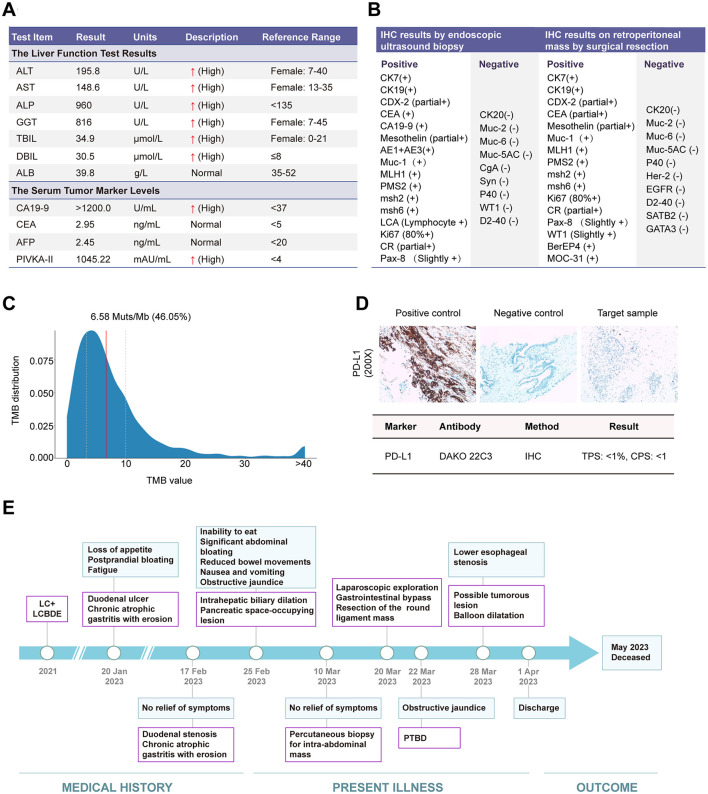
The clinical laboratory test results and histopathological assessment of the patient's intraperitoneal mass. **(A)** The liver function test results and serum tumor marker levels; **(B)** The immunohistochemistry (IHC) analysis from tissues obtained by endoscopic ultrasound biopsy and retroperitoneal mass resection; **(C)** The tumor mutational burden (TMB) result from the tumor biopsy specimen; **(D)** The programmed death-ligand 1 (PD-L1) expression level in the tumor biopsy specimen; **(E)** The timeline of events related to the patient's diagnosis and treatment.

The multidisciplinary consultation determined that the patient's inflammatory and autoimmune reactions subsequent to COVID-19 infection precipitated fibrotic changes in the retroperitoneal and pancreatic areas, as well as the hepatic hilum. These changes, in conjunction with the underlying polysplenia syndrome, collectively resulted in duodenal and biliary obstruction for the patient. Additional results from the positron emission tomography-computed tomography (PET-CT) exhibited mildly elevated metabolic activity in a space occupying lesion at the sites of the falciform and round ligaments. The liver function tests upon patient admission demonstrated severe hepatocellular damage, as indicated by markedly elevated levels of alanine transaminase (ALT) and aspartate transaminase (AST). Concurrently, dysregulation in bilirubin metabolism was observed, characterized by increased concentrations of total bilirubin (TBIL), direct bilirubin (DBIL), alkaline phosphatase (ALP), and gamma-glutamyl transferase (GGT; Figure 2A). Serum tumor marker analysis revealed a significant elevation of carbohydrate antigen 19-9 (CA19-9) and protein induced by vitamin K absence or antagonist-II (PIVKA-II; Figure 2A). To further ascertain the nature of the abdominal mass, the patient sequentially underwent percutaneous biopsy of the abdominal mass followed by resection of the round ligament lesion. Immunohistochemistry (IHC) analysis of the samples confirmed the tumor to be a poorly differentiated adenocarcinoma of pancreaticobiliary origin (Figure 2B). To elucidate the patient's tumor diagnosis and guide clinical medication use, biopsy tissue from the hepatic round ligament mass was analyzed using a comprehensive panel of cancer gene assays. Nonetheless, the testing outcomes failed to disclose any specific pathogenic variant types or identify any targetable DNA or RNA mutations available on the market. Additionally, tumor mutational burden (TMB) analysis (Figure 2C) and programmed death-ligand 1 (PD-L1) expression scoring did not indicate potential benefits from immunotherapy (Figure 2D).

Postoperatively, the patient was provided with nutritional support, anti-infective therapy, and electrolyte balance maintenance. Owing to the patient's poor tolerance, chemotherapy was not instituted. Percutaneous transhepatic biliary drainage (PTBD) was conducted to alleviate the obstructive jaundice. The patient later presented with dysphagia and vomiting; upper gastrointestinal double-contrast radiography revealed lower esophageal obstruction. Balloon dilation was performed to address these complications. The patient was discharged in a stable condition, maintaining basic total parenteral nutrition (TPN) treatment, and eventually passed away 2 months post-discharge (Figure 2E).

## 3 Discussion

Heterotaxy syndrome encompasses thoracoabdominal organ and vessel anomalies, categorized by symmetry (left/right isomerism) and situs types of solitus, inversus, or ambiguous (2, 6). Left isomerism, often manifesting as situs ambiguus, presents with polysplenia, bilateral bilobed lungs, bronchi anomalies, atrial isomerism, and IVC variations, alongside gastrointestinal rotational disorders (1, 2, 5, 6). In adults, heterotaxy syndrome is often incidentally identified through imaging studies such as PET-CT, X-ray, or ultrasound (2, 6–8). The syndrome's variable symptomatology, particularly in situs ambiguus (SA) forms, complicates diagnosis, as clinicians sometimes misuse the terms SA and situs inversus (SI) (6). Furthermore, significant variability in symptom severity and types is observed in the rare cases of heterotaxy syndrome occurring among closely related individuals.

Conditions frequently associated with heterotaxy syndrome in these familial cases include primary ciliary dyskinesia (PCD), Patau syndrome, Edwards syndrome, Ivemark syndrome, and Cumming type. The Cumming type is an autosomal recessive condition first described by Cumming et al. (9). They reported a case featuring campomelia, polycystic renal disease, cervical lymphocele, and polysplenia, with no evidence of heterotaxia in the pulmonary or cardiovascular systems. In 1997, Jeffrey et al. described a fetus with conditions akin to the Cumming type. Additionally, their case exhibited a unique constellation of anomalies including a short pancreas without a body and tail, as well as previously unreported features associated with the Cumming type such as abnormal lung lobation with bilateral left bronchial morphology, dextrocardia, total anomalous pulmonary venous return, a left superior vena cava, and a right aortic arch. These findings align with those observed in the polyasplenia spectrum. The authors suggested for the first time that the Cumming type should be broadened to encompass polysplenia with heterotaxia and proposed that Cumming syndrome might be considered another autosomal recessive condition linked to a laterality defect (10). Briefly, the Cumming type typically presents with tetramelic campomelia and a wide array of multi-organ anomalies (11). In contrast heterotaxy polysplenia primarily involves abnormal organ positioning with limb malformations being less common. Additionally, the Cumming type is classified as an autosomal recessive genetic disorder, whereas the genetic pattern of heterotaxy polysplenia is more complex and may be linked to multiple gene mutations. The genetic pattern is predominantly due to single-gene mutations, with inheritance modes including autosomal dominant (e.g., NODAL) and autosomal recessive (e.g., DNAH5 and CFC1) (12).

A range of genes critical for establishing left-right asymmetry during organogenesis—such as ZIC3, NODAL, LEFTY2, ACVR2B, CRYPTIC, CRELD1, NKX2.5, and CFC1—have been implicated in the familial clustering of heterotaxy-related conditions (4, 5). For instance, the CFC1 gene also plays a pivotal role in the regulation of left-right axis determination. The protein encoded by the CFC1 gene serves as a co-receptor on the cell membrane, binding to Nodal proteins and facilitating their interaction with the primary receptor, thereby activating the downstream Smad2/3 signaling pathway (13). During the early stages of embryonic development, CFC1 establishes the left-right asymmetry pattern by regulating the spatial distribution of Nodal signaling. It is also involved in the development of organs such as the heart, pancreas, and intestines during the mesodermal differentiation phase (14). Additionally, in the ciliated cells of the embryonic node, CFC1 may participate in the cilia-driven fluid flow, which influences the asymmetric distribution of left-right signaling molecules (15). Loss of CFC1 function leads to a reduction in Nodal signaling pathway activity, resulting in abnormal transmission of left-right axis signals in the embryo, which can cause polysplenia and disorders of organ symmetry (15). In clinical diagnostics, genetic testing for CFC1 can aid in identifying the hereditary etiology of visceral heterotaxia and differentiate it from phenotypes caused by teratogenic environmental factors, such as maternal diabetes or viral infections. Additionally, mutations in axonemal heavy chain dynein genes, including DNAH5, DNAH9, and DNAH11, have been identified as genetic factors contributing to PCD often accompanied by heterotaxy. The dynein heavy chains, which are essential components of the axonemes in cilia and flagella, and are involved in the movement of organelles and cellular signaling (16). Mutations in DNAH family members may lead to structural abnormalities in the ciliary axoneme, affecting ciliary beating and subsequently disrupting intracellular hydrodynamic sensing and signaling, resulting in the failure to establish left-right asymmetry (17). Moreover, ciliary dysfunction may cause abnormal cell migration, affecting the proper positioning and structure of the heart, spleen, and other organs. These mechanisms interact collectively to produce the phenotypic expression of heterotaxy syndrome (17). In our case, the patient's family showed no symptoms of heterotaxy syndrome, except for the patient's sister who had an asymptomatic SI form (Table 1). This suggests that genetic variation may underlie the phenotypic spectrum, warranting investigation into common variant genes as potential contributors to heterotaxy polysplenia syndrome.

**Table 1:** Phenotypic manifestations of heterotaxy syndrome and tumors in patients and their three-generation pedigree.

**Family members**	**Gender**	**Age**	**Heterotaxy**	**Polysplenia**	**Cancer status**
Proband	Female	36	SA	Y	Pancreaticobiliary adenocarcinoma
Father	Male	76	N	N	None
Mother	Female	77	N	N	Breast cancer
Sister	Female	40	SI	N	None
Brother	Male	35	N	N	None
Daughter	Female	5	N	N	None
Nephew 1	Male	7	N	N	None
Nephew 2	Male	11	N	N	None

Previous reports have indicated that up to 75% of all patients with heterotaxy syndrome diagnosed in childhood will experience mortality by age 5, and only 5%−10% of all patients are diagnosed as adults (6, 18). Most adult patients with heterotaxy polysplenia are diagnosed at the age of 50 years or older (6, 18). This is likely because serious cardiovascular issues rarely occur in heterotaxy polysplenia syndrome, which leads to delayed detection of the disease until more serious symptoms are discovered during radiological examinations or vascular interventions in mid to late adulthood. This finding is consistent with our patient who was characterized as having an SA with left isomerism polysplenia and congenital pancreatic insufficiency, and few to no cardiovascular issues other than the absence of a retrohepatic inferior vena cava. Thereby, the patient was not diagnosed until the age of 33 when she underwent laparoscopic cholecystectomy.

There have been relatively few reports confirming a predisposition to hepatobiliary conditions in association with heterotaxy polysplenia syndrome. During embryonic development, the final position of the common bile duct is determined by the coordinated rotation of the pancreas, midgut, and spleen. These organs are believed to share a common developmental origin. This partially explain why polysplenia is frequently accompanied by structural abnormalities of the biliary tract, intestines, and pancreas. For instance, biliary atresia occurs in up to 10% of heterotaxy polysplenia cases, and intestinal malrotation occurs in up to 70% (19). Anomalies in pancreatic structure, such as an annular pancreas, can compress the encircled major duodenal papilla, leading to duodenal atresia and obstruction. This consequently causes abnormal bile flow within the common bile duct, impeding normal bile excretion and resulting in choledocholithiasis (5, 7). In our case, the patient exhibited anatomical structural abnormalities such as congenital pancreatic insufficiency and duodenal obstruction. These findings provide support for the concept that developmental homology is an important cause of the susceptibility to anatomical structural abnormalities of abdominal organs in patients with polysplenia syndrome. Regarding the patient's biliary anatomy, there is no record of congenital biliary abnormalities, such as biliary atresia, and the patient has not previously undergone a Kasai procedure. Two years prior to admission, the patient underwent a laparoscopic cholecystectomy combined with exploration and stone extraction from the common bile duct. The MRCP revealed circumferential compression of the common hepatic duct by the enlarged pancreatic head, which was likely the cause of the intrahepatic ductal dilatation. Additionally, the retroperitoneal mass compressed the hepatic hilum, leading to stenosis of the hilar bile ducts. The impaired bile flow resulted in the patient's presentation with mild jaundice of the skin and mucous membranes. Liver function tests indicated significant liver damage, partially attributable to chronically elevated biliary pressure. During the hospitalization, the patient underwent a gastrojejunostomy and excision of a round ligament mass; however, the biliary obstruction was not alleviated. Consequently, the patient was treated with PTBD to address the obstructive jaundice.

**Figure 3:**
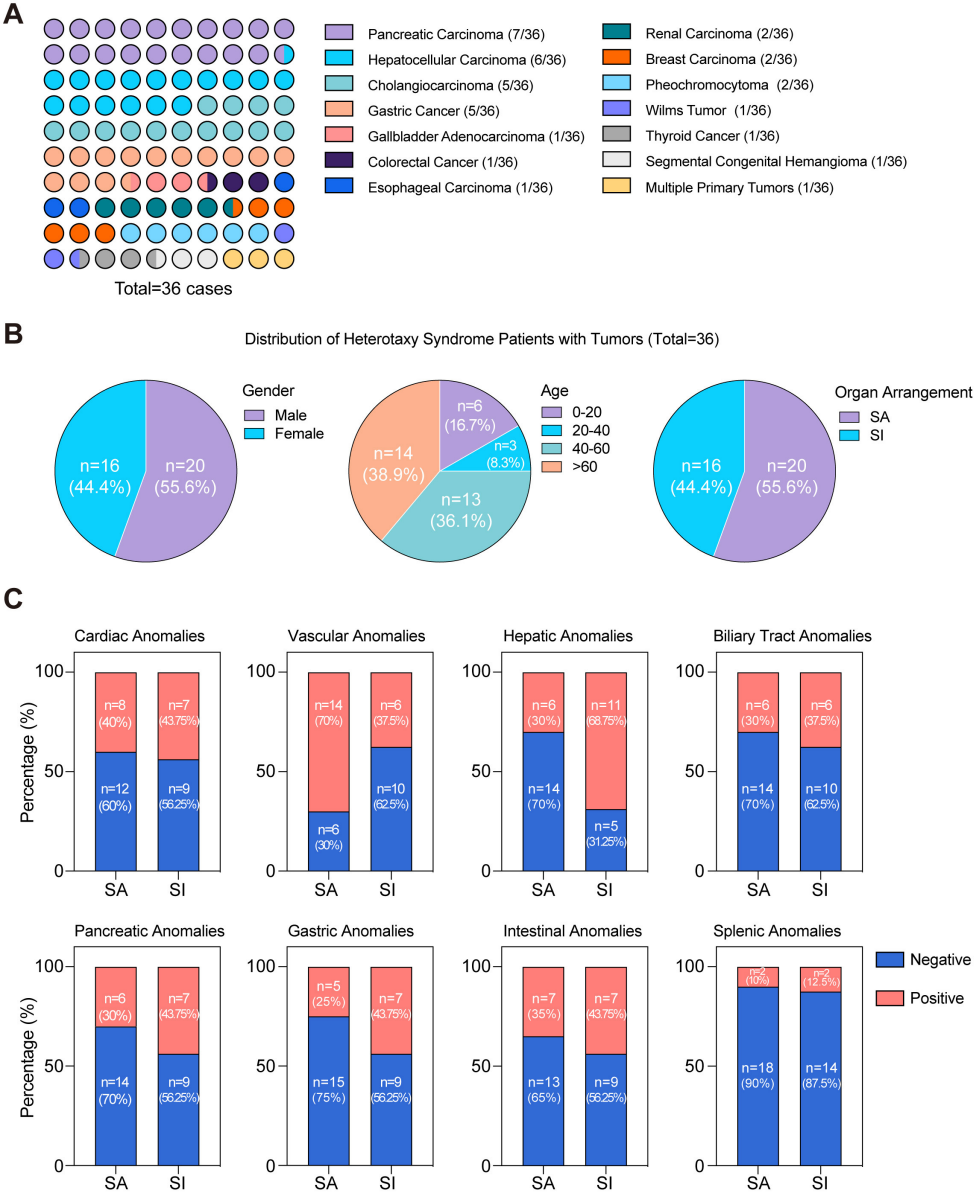
Distinct distribution characteristics in heterotaxy syndrome cases with tumors (1984–2023). **(A)** Distribution of cancer types in heterotaxy polysplenia syndrome patients; **(B)** Distribution characteristics of heterotaxy syndrome with tumors stratified by gender, age, and organ arrangement; **(C)** The proportion of organ abnormalities in two types of visceral arrangement pattern among 36 cases.

To elucidate the underlying links between heterotaxy syndrome and the genesis of malignant tumors, we performed a search in PubMed for all case reports, employing the search query “(‘polysplenia' or ‘heterotaxy') and ‘cancer'.” Between 1984 and 2023, a total of 621 cases of heterotaxy syndrome or polysplenia were reported, with 36 cases (~5.8%) diagnosed with malignant tumors. Notably, these cases encompassed 14 different cancer types, with a predominance of hepatobiliary and gastrointestinal malignancies, representing 72.2% of the total cases (26/36) (Figure 3A). Besides, gender distribution analysis revealed a higher proportion in male patients (Figure 3B). The age distribution pattern showed a higher prevalence among patients over 40 years of age (Figure 3B). Notably, two infants under 1 year of age were included: one diagnosed with Wilms tumor (20) and the other with segmental congenital hemangioma (21). Regarding heterotaxy syndrome, 20 cases had situs ambiguus (SA), and 16 cases had situs inversus (SI) (Figure 3B). These observations underscore the diversity of the heterotaxy patients with malignant tumors. Additionally, we analyzed the proportion of organ abnormalities in two types of visceral arrangement pattern (SA and SI) and observed a higher prevalence of organ anomalies in situs inversus group (Figure 3C). This result indicates that the organ-specific anomalies are more frequently associated with the atypical arrangement observed in situs inversus. Further analysis revealed that a single organ can manifest a variety of abnormalities. For instance, pancreatic anomalies (13/36) demonstrated a range of conditions, including annular pancreas, pancreatic truncation, absence of the pancreatic head or tail, and dorsal pancreatic agenesis. Interestingly, these pancreatic anomalies were observed in seven patients with pancreatic carcinoma and in nine patients with other cancer types. Two patients with a family history of situs inversus were identified, and both were diagnosed with pancreatic carcinoma (22). The nine cases included two cases of hepatocellular carcinoma (18, 23), three of cholangiocarcinoma (24–26), two of gastric carcinoma (27), one of colorectal cancer (28), and one of thyroid carcinoma (29). Further investigation revealed that seven patients with pancreatic carcinoma underwent either separate duodenectomy, total pancreatectomy, or pancreatoduodenectomy surgical treatment, and pathological analysis of the tumor specimens revealed stage II or III adenocarcinoma. Six out of the seven patients did not require additional postoperative treatment and were discharged in good condition, whereas one patient received standard adjuvant gemcap treatment after surgery. Follow-up CT scans of all of these patients performed from 6 months up to 2 years postsurgery showed no signs of recurrence (8, 30, 31). Unfortunately, the unresectable nature and unspecified origin of the tumor in our patient greatly hindered the availability of treatment options. These findings indicate a potential correlation between organ anomalies associated with heterotaxy and the tumorigenesis in congenitally malformed organs, particularly among patients with pancreatobiliary malignancies.

In summary, our case report enhances the current understanding of the association between heterotaxy polysplenia syndrome and malignant tumors, particularly in patients with a strong familial predisposition. We emphasize the necessity of integrating effective clinical diagnostic information with a patient's familial and genetic background as a standard procedure. This approach is essential for achieving effective clinical interventions and contributes valuable insights for future research.

## Data Availability

The raw data supporting the conclusions of this article will be made available by the authors, without undue reservation.
